# Reducing Emergency Department (ED) Boarding Time Through Lean Redesign of the ED-to-Inpatient Admission Pathway: A Single-Center Quality-Improvement Study

**DOI:** 10.7759/cureus.109766

**Published:** 2026-05-27

**Authors:** Sallehah M Farok, Khalid A Ateyyah, Ameera J Almotairi, Babatunde N Thomas, Othman M Alkhaibari, Ameen H Alhazmy, Waleed A Alharbi

**Affiliations:** 1 Emergency Department, King Faisal Specialist Hospital and Research Centre, Madinah, SAU; 2 Medicine/Emergency Medicine, Taibah University, Madinah, SAU

**Keywords:** emergency department efficiency, emergency departments (eds), length of stay, overcrowding, patient outcomes, patient wait times, process improvement interventions

## Abstract

Background

Boarding inflates emergency-to-inpatient throughput time. We implemented a change package, pulling bed decisions upstream, disciplining handoffs, and removing a large diagnostic gate in a tertiary emergency department (ED), and evaluated its operational impact.

Methodology

We conducted a single-center, pre-post, quality-improvement study of adult ED admissions from January to August 2023. The baseline phase included admissions from January to March 2023, and the post-intervention phase included admissions from April to August 2023 after implementation of the Lean redesign on April 1, 2023. The primary endpoint was patient-level total admission cycle time, which was captured electronically from the electronic health record (EHR) and calculated as the number of minutes from admission order to inpatient bed arrival. Value stream mapping was utilized as a complementary process diagnostic tool to illustrate typical pathway lead times, categorize value-added and non-value-added activities, and determine process efficiencies. Map-based step medians were not considered to be directly interchangeable with aggregate patient-level cycle times, but rather as a diagnostic summary.

Results

Total admission cycle time as reported by EHRs (baseline, N = 217; post-intervention, N = 218) showed a reduction from 390 to 175 minutes (-215 minutes; 55% relative reduction). Non-value-added time fell from 290 to 90 minutes (-200 minutes; -69%) while value-added time saw a more modest change from 100 to 85 minutes (-15%). The process efficiency rose from 25.6% to 48.6% (+23.0 percentage points; ~+90% relative). Process-map results indicated that there was an improvement in the direction, and that three of the most significant sources of non-value-added time were diagnostic waiting, bed coordination, and transfer delays.

Conclusions

This single-center, pre-post, quality-improvement study found that a Lean redesign of the ED-to-inpatient admission pathway was linked to a clinically meaningful decrease in the admission cycle time and an increase in process efficiency. The results indicate that there may be value in pursuing earlier bed coordination, risk-based diagnostic gating, and standardizing handoffs as targets for future controlled or multisite evaluation, but not causal evidence.

## Introduction

Emergency departments (EDs) are 24/7 safety-net services that now shoulder steadily rising demand, case-mix complexity, and non-urgent presentations, straining flow and safety worldwide [1]. Recent national snapshots illustrate the scale: in the United States, the 2022 ED visit rate was 47 per 100 people (≈155 million visits), and in 2016, only 39% of patients were seen by a clinician within 15 minutes [2]; in Canada, unscheduled ED visits reached ~15.5 million in 2023-2024 with prolonged stays for admitted patients [3]. These trends meet with well-described input, throughput, and output constraints in emergency care. Access block is a central output constraint, which is the delayed transfer of patients needing hospitalization to an available hospital bed. If this happens, admitted patients are held in the ED until they are admitted to a ward, which is known as “inpatient boarding.” Access block and boarding decrease ED treatment capacity, increase waiting times, and are a significant contributor to crowding. Recent studies that use NEDOCS-based crowding assessment and Lean process-improvement methods demonstrate that delayed inpatient transfer is a measurable and modifiable driver of ED crowding and boarding time [4-6].

ED crowding is a result of multiple patient, operational, and system factors that interact with one another. Patient factors include a large number of arrivals, non-urgent presentation, frequent presentations to ED, and increasing complexity of cases. Operational contributors are staffing constraints, porterage constraints, delays in consultations, delays in completion of routine orders before transfer, and delays in turnaround time for diagnostic services (laboratory and imaging). System-level contributors include constrained inpatient bed capacity, delayed bed readiness, and access block, which together prolong ED length of stay (LOS), increase left-without-being-seen (LWBS) rates, and heighten the risk of adverse events [5,7]. Systematic reviews and contemporary analyses link ED boarding and prolonged ED LOS to patient harm and operational degradation, underscoring the need for local, process-aware remedies [8].

These pressures are also prominent in Saudi Arabia, where rising emergency care demand, substantial ED use for non-emergency conditions, and seasonal mass gathering surges during Hajj and Umrah place additional strain on regional referral hospitals [9,10].

A baseline review of ED admissions at King Faisal Specialist Hospital and Research Centre (KFSHRC) in Madinah was conducted in January-March 2023, revealing that the ED to in-patient transfer pathway has a number of delays, including receiving-unit acceptance, bed readiness, completion of pre-transfer orders, porterage, and COVID-19 testing requirements. To address this, we started by using a value stream mapping (VSM) approach to identify value-added and non-value-added time within this pathway and then began a Lean redesign process to achieve better compliance with the departmental ≤2-hour admission boarding-time standard. The evaluative question was: whether the implementation of this redesigned pathway on April 1, 2023, correlated with decreased admission cycle time and increased process efficiency compared to the baseline period?

## Materials and methods

Study design, setting, and reporting framework

The study was a pre-post quality-improvement study in the adult ED of KFSHRC, Madinah, Saudi Arabia. The project was reported using SQUIRE 2.0 to enhance transparency about the local problem, the intervention rationale, contextual factors, measurement strategy, and analytic approach.

The study period was from January to August 2023. The baseline phase was from January 1 to March 31, 2023, where the current admission pathway was reviewed and mapped. The post-intervention phase included admissions from April 1 to August 31, 2023, following implementation of the Lean redesign of the ED-to-inpatient admission pathway.

Local problem and improvement rationale

The improvement initiative focused on the delays in getting patients to an inpatient bed after the decision to admit a patient was made. An initial audit of the ED-to-inpatient transfer pathway revealed delays in receiving-unit acceptance, inpatient bed availability, completion of routine pre-transfer orders, portering and transfer coordination, and COVID-19 testing before inpatient placement. These delays added up to significant amounts of non-value-added time in the admission pathway and helped inform the pathway-focused Lean redesign.

The project aimed to increase adherence to the departmental ≤2-hour admission boarding-time target by decreasing avoidable delays following the admission decision and enhancing the coordination between ED, inpatient, and bed-management teams.

Study population and eligibility criteria

The study population consisted of the adult population admitted to an inpatient ward from the ED during the study period. Encounters were categorized by the following study phases: baseline (January 1-March 31, 2023) and post-intervention (April 1-August 31, 2023).

Eligible encounters were ED-to-inpatient admissions that used the standard pathway for ward transfer and had the necessary time stamps to assess admission flow. Encounters were excluded if they were trauma activations, cardiac arrests, direct intensive care unit admissions that did not go through the routine ward-transfer process, inter-facility transfers, or records with missing or implausible key timestamps.

Process analysis using value stream mapping

A structured VSM approach was used to characterize the admission pathway from admission order signature to arrival in an inpatient bed. The process analysis distinguished between value-added time and non-value-added time and was used to develop present-state and post-intervention pathway maps. Mapping was done following the standard Lean healthcare and VSM conventions [11,12]. Process analysis distinguished between: (1) Value-added time: clinically necessary and operationally essential activities that directly contributed to patient care or transfer readiness, including physician assessment, necessary diagnostic steps, and activities to prepare for transfer.* *(2) Non-value-added time (NVAT): waiting, queueing, redundant handoffs, delays due to porterage or bed availability, and other delays that did not directly contribute to the advancement of patient care or completion of the transfer.

The analysis included a present-state map of the baseline pathway and a future-state/current-state map of the redesigned pathway following the implementation of the interventions. Electronic health record (EHR) timestamps, bed-management and laboratory system timestamps (when available), and direct process observations to clarify workflow sequence and operational handoffs were used to inform mapping.

Intervention development

A hybrid nominal group technique was used to guide the development of the intervention. This involved independent idea generation, round robin listing, clarification, clustering of proposed solutions, and multi-criteria scoring according to the expected impact, feasibility, safety, and cost. Systems improvement personnel, the Clinical Command Center/admissions team, ward nursing leadership, and ED clinicians comprised the multidisciplinary improvement team.

The intervention rationale was to address the major bottlenecks identified in the baseline mapping and delay analysis. The baseline Pareto assessment was based on the following four categories of drivers: (1) receiving-unit delays, (2) bed-readiness issues, (3) requirements for COVID-19 swab/testing, and (4) completion of routine patient orders in the ED before transfer.

Iterative Plan-Do-Study-Act cycles were used to validate and refine the candidate countermeasures before implementation, based on the VSM results. Following this refinement phase, the final intervention package was implemented as an integrated multi-component bundle beginning April 1, 2023, rather than as sequential components intended for separate effect attribution. The final intervention package comprised: (1) pull-based bed management, in which inpatient placement coordination was initiated earlier after the admission decision to reduce downstream waiting; (2) standardized policy education, including reinforcement of the ≤120-minute decision-to-bed target among ED and inpatient teams; (3) optimization of ED triage and fast-track processes to reduce avoidable internal congestion affecting downstream admission flow; (4) improved porterage escalation procedures to address transport-related delays once patients were ready for transfer; and (5) replacement of routine COVID-19 testing as a universal transfer gate with a more risk-based testing approach, consistent with contemporaneous infection-control requirements.

Monitoring of implementation was conducted using pragmatic process-control activities, not a formal component-specific fidelity score. These activities included daily in-service debriefing, nursing champions within the units to reinforce policy expectations, monthly review of admission flow data, introduction of standardized admission boarding time tracking, and tracking the admission delay patterns associated with ward acceptance, bed availability, and transfer progression. The study was not intended to separate the contribution of each component of the intervention because the intervention was delivered as a package of quality improvement (Table 1).

**Table 1:** Operational components, implementation timing, and monitoring of the intervention bundle. ED = emergency department; PDSA = Plan-Do-Study-Act; VSM = Value Stream Mapping

Intervention component	Operational change introduced	Implementation timing	Implementation monitoring
Pull-based bed management	Earlier initiation of inpatient placement coordination after the admission decision	Implemented with the April 1, 2023, intervention bundle	Review of admission-flow intervals and bed/ward-related delay patterns
Standardized policy education	Reinforcement of the ≤120-minute decision-to-bed target among ED and inpatient teams	Implemented with the April 1, 2023, intervention bundle	Daily in-service debriefings, nursing-champion dissemination, and policy reinforcement
ED triage and fast-track optimization	Workflow adjustments intended to reduce avoidable internal congestion affecting downstream admission flow	Implemented with the April 1, 2023, intervention bundle	Discussed during PDSA refinement and daily barrier review
Porterage escalation procedures	Escalation of transport-related delays after patients were ready for transfer	Implemented with the April 1, 2023, intervention bundle	Monitoring of transfer-progression delays during admission-flow review
Risk-based COVID-19 testing	Routine COVID-19 testing was no longer used as a universal transfer gate; testing became more risk-based	Implemented with the April 1, 2023, intervention bundle	Review of COVID-19-related delay patterns in VSM and post-intervention pathway assessment

Measurement framework and data sources

The primary outcome was the weekly median patient-level EHR-derived admission cycle time, which was measured as the time in minutes between the placement of the admission order and the patient’s arrival in an inpatient bed. To study the temporal dynamics of the primary outcome over the course of the study, and to make the evaluation more consistent with quality-improvement methodology, weekly medians were chosen. Medians were also computed at the phase level as baseline and post-intervention summary measures.

Secondary operational measures were selected at segment-specific intervals within the admission pathway, e.g., time related to COVID-19 testing, bed coordination, transfer preparation, and ward acceptance. These measures were used to place the delays in context and to track the pathway following the implementation of the intervention.

Process diagnostics based on VSM were evaluated separately from the main outcome based on EHR. These included: (1) cumulative value-added time; (2) cumulative non-value-added time; (3) total mapped lead time; and (4) process efficiency, defined as value-added time/total mapped lead time.

These process-map metrics were used as diagnostic summaries of the typical pathway and were not treated as directly interchangeable with aggregate patient-level EHR-derived admission cycle times.

Timestamp crosswalk and operational measurement

A timestamp crosswalk was applied to sync up the major steps in the workflow with the primary data sources to standardize measurement across the pathway. The pathway started at the point of admission order placement and ended at the point of arrival to an inpatient bed. The intermediate steps were ordering of COVID-19 testing, collection of the specimen, the specimen being received in the lab, verifying COVID-19 test results, submission of the bed request, bed assignment, initiation of environmental services, completion of environmental services, signaling of bed readiness, and, if applicable, arrival of the consulting clinician, and the ED departure. These timestamps were taken from the EHR, laboratory information system, bed-management system, environmental services records, and admission-discharge-transfer documentation, depending on the process step.

Required pathway timestamps were checked for completeness and chronological validity before analysis. Encounters with missing key timestamps or implausible/non-sequential time values were excluded according to the predefined eligibility criteria. This data-quality review was performed to ensure that the admission cycle-time outcome and pathway-segment calculations reflected valid ED-to-inpatient transfer sequences.

Statistical analysis

The baseline and post-intervention periods were descriptively compared. Medians and descriptive distributions were used to summarize the primary outcome and pathway metrics. The absolute change and relative percentage change were determined for admission cycle time, value-added time, non-value-added time, and total lead time. The changes in process efficiency were reported as absolute percentage points as well as relative percentage changes. The total admission cycle time was reduced as the proportion of reduction from baseline to the post-intervention period. Segment-specific median times were analyzed to understand contributors to delays observed and to interpret the operational effect of the intervention bundle. No causal inference was intended from the pre-post quality-improvement design; findings were interpreted as associations between implementation of the redesigned pathway and observed changes in admission flow metrics.

Ethics oversight and operational governance

The study was conducted in accordance with the Declaration of Helsinki and approved by the Research Ethics Committee (REC) of KFSHRC (approval number C1745M/14/45 and date of approval: October 9, 2023). Consent was waived by the REC for this quality-improvement study. The shift from COVID-19 testing as a transfer gate to COVID-19 testing as a risk-based test was done in conjunction with, and with the approval of, the appropriate hospital infection-control and operations leadership, based on the hospital’s current policy.

## Results

Cohort flow

**Figure 1:**
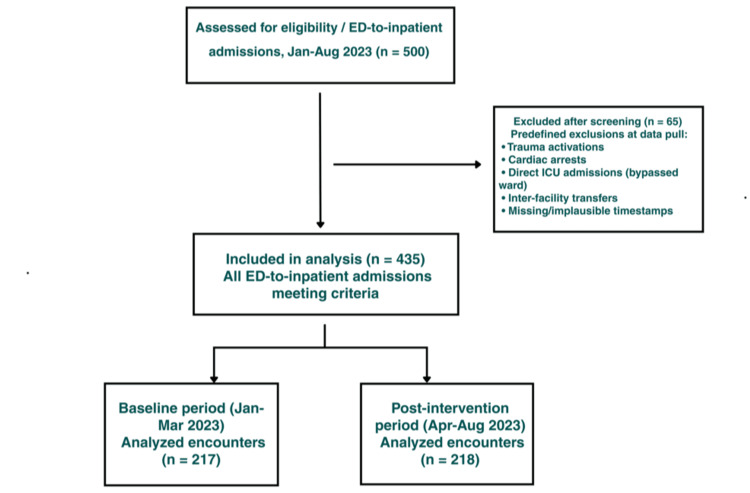
Cohort assembly diagram. ED = emergency department; ICU = intensive care unit

A total of 500 adult ED-to-inpatient admission encounters were identified during the January-August 2023 study period. After application of the predefined exclusion criteria, 65 encounters were excluded, leaving 435 encounters in the analytic cohort. Of these, 217 encounters occurred during the baseline period from January to March 2023, and 218 occurred during the post-intervention period from April to August 2023 (Figure 1).

Admission cycle time and process-efficiency outcomes

The median EHR-derived admission cycle time (time between the placement of the admission order and arrival in an inpatient bed) was 390 minutes in the baseline period and 175 minutes after the intervention bundle was implemented. This was a 215-minute absolute and 55.1% relative decrease. The complementary process-efficiency analysis revealed a reduction of value-added time from 100 to 85 minutes (-15%) and a reduction of non-value-added time from 290 to 90 minutes (-69%). This led to an improvement in process efficiency from 26% to 49% (23 percentage point absolute improvement, 89.4% relative improvement) (Table 2).

**Table 2:** Change in efficiency before and after the intervention. Flow efficiency is value-added time/total time. pp = percentage point

	Before intervention	After intervention	Absolute change	Relative change (%)
Value-added activity time (minutes)	100	85	-15	-15
Non-value-added activity time (minutes)	290	90	-200	-69
Total cycle time (minutes)	390	175	-215	-55.1
Flow efficiency	26%	49%	+23 pp	89.4

Cycle time and efficiency before and after the intervention

All (n = 435) patients who were admitted through the emergency room were included during this project period. Of these, the number of admission delays in January, February, and March 2023 was 31, 31, and 24, respectively. At baseline, the total admission cycle, from admission order to arrival in an inpatient bed, required 390 minutes, comprising 100 minutes of value-added work and 290 minutes of non-value-added time (Table 3). After implementing the change package, total time fell to 175 minutes (-215 minutes; 55% reduction), driven largely by compression of waste: non-value-added time decreased from 290 to 90 minutes (-69%), while value-added time decreased modestly from 100 to 85 minutes (-15%). Process efficiency (value-added/total time) therefore increased from 26% to 49%, a +23-percentage-point (~90% relative) improvement.

**Table 3:** Exemplar map block ↔ timestamp crosswalk and mapping conventions (lean healthcare). T0 denotes the start of the admission pathway. Turnaround time (TAT) was calculated using the relevant timestamps shown in the table. ADT = admission, discharge, and transfer; EHR = electronic health record; EVS = environmental services; LIS = laboratory information system

Map block (label)	Operational definition	Primary timestamp/Field	System of record	Use in analysis
Admission order placed	Admission decision documented in the EHR	admission_order_time	EHR (orders)	Defines pathway start (T0)
COVID test ordered	COVID-19 laboratory order entered in the EHR or LIS	lab_order_time	EHR/LIS	Opens the testing gate
Specimen collected	Sample collected at the bedside	specimen_collection_time	LIS/EHR flowsheet	Defines the start of laboratory turnaround time
Lab received	Specimen received by the laboratory	specimen_received_time	LIS	Measures laboratory queue or dwell time
Result verified	Test result verified and released to the EHR	result_verified_time	LIS/EHR	Defines the end of testing gate; used to calculate laboratory turnaround time
Bed request sent	Bed request submitted to bed management	bed_request_time	Bed management system	Defines the start of the bed orchestration process
Bed assigned	Specific inpatient bed allocated to the patient	bed_assigned_time	Bed management system	Triggers environmental services/page workflow; used to measure assigned-to-ready lag
EVS start	Bed cleaning initiated	evs_start_time	EVS/EHR interface	Measures cleaning latency
EVS complete	Bed cleaning completed	evs_complete_time	EVS	Measures cleaning duration
Bed ready signal	Bed marked as ready for patient placement	bed_ready_time	Bed management system	Defines end-of-bed-readiness lag
Consult arrival (if required)	First consulting clinician arrives at bedside	consult_start_time	EHR (consult note/arrival)	Assesses parallel work and tail-risk delays
ED departure	Patient physically leaves the emergency department	ed_departure_time	EHR (ADT)	Defines the start of the transport/transfer phase
Inpatient bed arrival	Patient arrives on the inpatient unit	inpatient_arrival_time	EHR (ADT)	Defines admission-flow endpoint

Process-map interpretation

**Figure 2:**
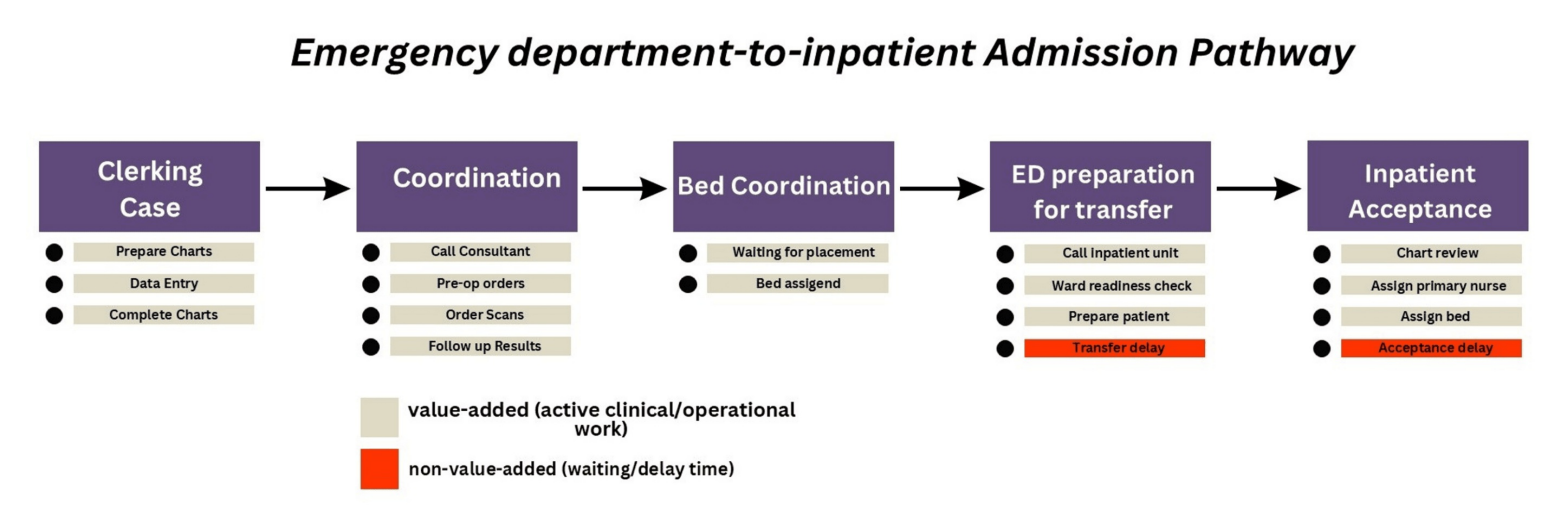
Overview of the pathway from patient review in the emergency department to transfer to an inpatient bed. Times represent median values across the cohort (actual mapping). ED = emergency department

Process maps corroborated where these gains occurred. In the pre-intervention (actual) pathway, the lead time was 260 minutes with 100 minutes value-added; the predominant bottleneck was the COVID-19 test/result loop, which contributed a 120-minute wait and accounted for roughly 46% of the total pathway time (Figure 2). In the post-intervention (current) pathway, the COVID-19 gate was removed/streamlined, bed assignment was advanced, and the pre-transfer checklist was condensed, reducing lead time to 90 minutes with 85 minutes value-added, indicating that nearly all saved time arose from eliminating non-value-added steps (Figure 2). These map-level changes are concordant with the aggregate cycle-time and efficiency improvements observed in Table 3. As map step-times are medians of a “typical path,” totals will be lower than EHR-derived cycle time.

Pareto profile of delays across months

**Figure 3:**
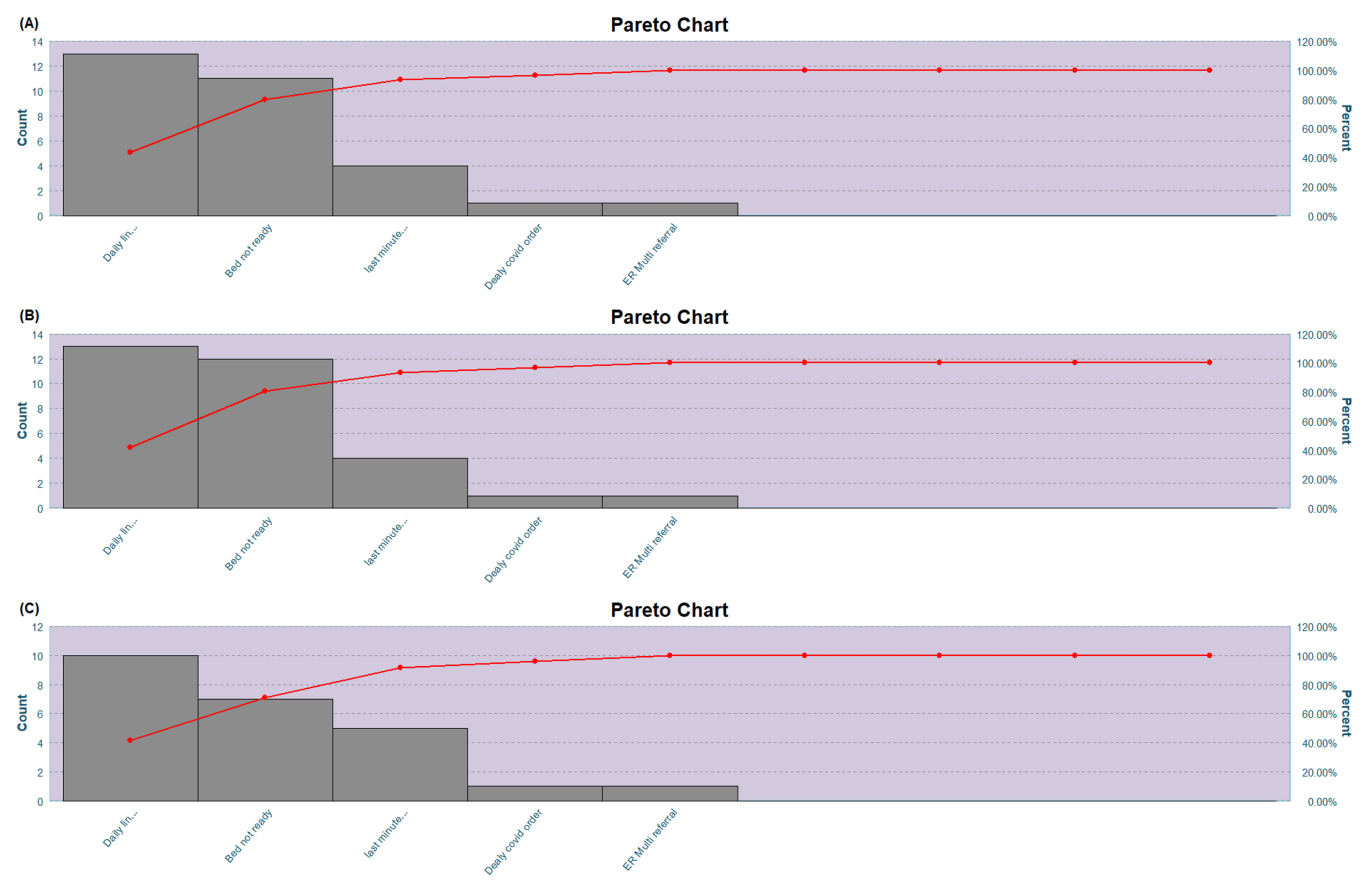
Pareto charts demonstrating the root causes of recorded delay events within the emergency department of King Faisal Specialist Hospital and Research Centre in January (A), February (B), and March (C) 2023. Bars represent the number of recorded delay events in each category, while the cumulative line represents the percentage contribution of each category to the total monthly delay-event count.

**Figure 4:**
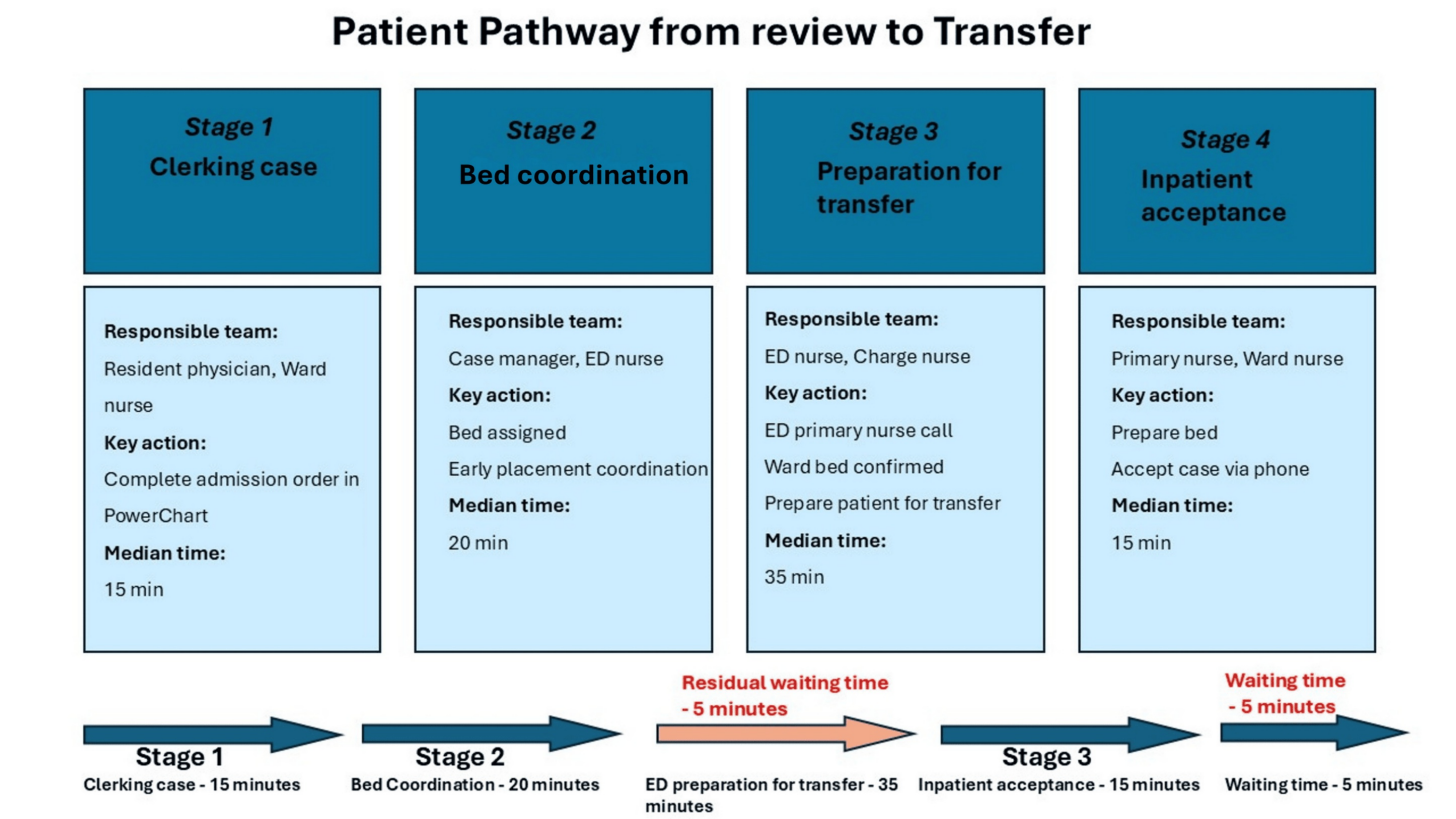
Overview of the pathway from patient review in the emergency department to transfer to an inpatient bed. Times represent median values across the cohort (current mapping). ED = emergency department

Monthly Pareto charts demonstrated a stable, highly concentrated error structure (Figures 3A-3C). Total inefficiency events numbered 31 in January (Figure 3A), 31 in February (Figure 3B), and 24 in March (Figure 3C). Across all three months, the leading category, “Delay in receiving unit,” consistently accounted for about 42% of events. When combined with “Bed not ready,” the cumulative share reached 77-81% in January-February and approximately 71% in March; adding the third-ranked “Last-minute orders” category brought the cumulative coverage to ~90-94% each month. Thus, targeting the top two to three contributors was expected to mitigate roughly nine out of ten observed delays. March showed a lower event volume with a slightly flatter distribution (top three ≈ 10/7/5 events), suggesting partial improvement in bed readiness with persistent upstream delays, changes that align with the pathway simplifications depicted in Figure 2 and Figure 4.

Alignment of drivers, actions, and process control targets

The improvement strategy was anchored to a SMART aim to increase compliance with the ≤2-hour admission-boarding time by 50% by August 2023 and sustain through December 2023, and to three system drivers mapped to concrete actions (Table 3). First, policy awareness gaps were closed through in-service education for ED and inpatient teams, designation of nurse champions to cascade updates, and explicit engagement of ED physicians and consultants. Second, deficiencies in measurement and statistical visibility were addressed by instituting an ED coordinator feed of monthly admissions, assigning two nurses to capture admission boarding time for every patient, and maintaining a standardized admission boarding time registry. Third, task-coordination problems were mitigated by monitoring time to first evaluation by the consulting resident, tracking multiple referrals within the ED, and auditing ward receipt times and bed availability.

Process control targets and alignment with observed flow improvements

Process control targets were specified to keep improvement work on course: ≤15 minutes from decision-to-admit to the physician admission order, ≤30 minutes for patient preparation for transfer, and daily in-service debriefings to reinforce practice and surface barriers (Table 4). The observed 55% reduction in total cycle time and the rise in efficiency from 26% to 49% are consistent with meeting these intermediate targets and with the observed collapse of non-value-added time on the pathway maps (Table 3; Figures 3, 4). In combination with the Pareto concentration of delays (Figure 3A-3C), these findings indicate that a driver-linked intervention bundle focused on policy clarity, real-time measurement, and coordinated handoffs can materially shorten admission cycles and position the system to meet the ≤2-hour boarding KPI. Quantitative monthly compliance rates will further define the magnitude and durability of target attainment.

**Table 4:** Key drivers and interventions to improve admission boarding time. SMART aim: To increase compliance with the emergency department (ED) admission boarding time target of ≤2 hours by 50% by the end of August 2023 and sustain this improvement through December 2023, in accordance with hospital policy and key performance indicators.

Driver	Intervention
Limited awareness of the admission policy	Conduct in-service sessions for ED staff on the admission time policy and key performance indicator targets
Limited awareness of the admission policy	Designate one nurse from each unit to disseminate policy information to ward nurses
Limited awareness of the admission policy	Reinforce policy expectations among ED physicians and consulting teams
Limited availability of data to evaluate admission delays	Engage the ED coordinator to provide monthly data on total admissions through the ED
Limited availability of data to evaluate admission delays	Assign two nurses to record admission boarding time for each eligible patient
Limited availability of data to evaluate admission delays	Establish a standardized admission boarding time tracking sheet in Excel
Poor coordination among members of the healthcare team	Monitor the time taken for the consulting medical resident to assess the patient
Poor coordination among members of the healthcare team	Monitor delays related to multiple referrals in the ED
Poor coordination among members of the healthcare team	Monitor bed availability and the time taken by inpatient wards to receive admitted patients

## Discussion

This quality-improvement program delivered a rapid, system-level shortening of the admission pathway from the ED to inpatient beds. The total cycle time fell by 55% (390 to 175 minutes) with a near doubling of efficiency (26% to 49%), and VSM showed that most of the time saved came from eliminating non-value-added waits rather than compressing clinical touch time. Two observations are central. First, the Pareto structure of delays was stable; in three consecutive months, “Delay in receiving unit” and “Bed not ready” accounted for 70-81% of events, with “Last-minute order” bringing cumulative coverage to 90-94%. Second, the largest single bottleneck in the baseline pathway was an avoidable diagnostic gate (a COVID-19 testing/result loop contributing ~120 minutes of waiting), while downstream bed coordination and pre-transfer steps contributed the next largest delays. After removing/streamlining the gate and pulling bed assignment earlier, the current map shows a lead-time of 90 minutes with 85 minutes of value-added work, indicating that the intervention mainly attacked the “hidden factory” of rework and waiting.

Multiple global, regional, and local studies are concerned with emergency care practice, with many controversial and contradicting results [13-15]. The headline effect in our project, a reduction in the admission cycle from 390 to 175 minutes (-55%) with efficiency rising from 26% to 49%, is concordant with what the emergency medicine literature predicts when boarding pressure and handoff friction are relieved. Foundational syntheses have long linked ED crowding and boarding to treatment delays, prolonged LOS, ambulance diversion, and worse outcomes. Hoot and Aronsky’s systematic review summarized this multifaceted harm landscape almost two decades ago, and subsequent updates have kept the signal directionally consistent [16]. More recent observational cohorts have sharpened the clinical stakes; spending the night boarding in the ED is associated with higher in-hospital mortality in older adults, and the risk of delirium increases with boarding duration, effects that are biologically plausible and operationally important [17].

Our maps show the main pre-intervention delay was a 120-minute COVID-19 testing loop. This matches pandemic reports: how testing is organized matters as much as testing itself. Point-of-care/rapid tests shortened ED LOS and isolation time, while universal testing at low prevalence prolonged admissions without added diagnostic yield [18]. Post-intervention, we “de-gated” COVID-19 testing, tightened indications, and sped up turnaround, cutting mapped lead time by 170 minutes (260 to 90 minutes). This aligns with ED studies showing hour-level savings when slow diagnostic gates are replaced with faster, targeted ones [19].

Second, bed orchestration matters. Studies show that managing downstream capacity signals, not just adding beds, drives flow. McCarthy et al. found that crowding and boarders prolong ED LOS across all acuity levels, and White et al. showed each additional boarder lengthens the stay of every ED patient, even those discharged [20,21]. Reviews show that ED boarding prolongs hospital stay, typically by ≥1 day and up to ~3 days for long boarders, and creates spillovers: more elopements and system-wide delays [4]. Our Pareto charts show the same pattern locally: “Delay in receiving unit” and “Bed not ready” account for 70-81% of defects each month; adding “Last-minute orders” brings coverage to ~90-94%. The stability points to structural coordination problems, not one-offs, precisely where real-time visibility and standardized handoffs help. Although randomized controlled trials of bed dashboards are limited, quality-improvement studies and a recent hospital-dashboard review find that electronic whiteboards/real-time displays improve situational awareness and timeliness, especially when governance and workflows are co-designed with clinical teams [22].

Third, standardized handoffs matter in the volatile window between admission decision and transfer. The Joint Commission flags ED boarding as a safety risk that harms patients and clinicians. ED-adapted I-PASS tools cut information loss at shift change and, when built into the EHR, make ownership and tasks explicit during boarding [23]. In our project, nursing/physician engagement and daily debriefs served as a basic form of standardization. Formalizing this should further cut the small residual non-value-added time after the intervention. While there are some boarding models that are dedicated and protocolized, which can help reduce some of the negative impacts of extended ED stays, these are resource-intensive and not widely available across institutions [24]. Thus, the more generalizable approach is to reduce avoidable boarding delays by tackling the biggest operational bottlenecks.

Another tension is universal testing. In uncertain times, many hospitals screened everyone for COVID-19 before admission. The goal was safety, but it slowed the system. In the study by Sangal et al., universal ED testing made admission LOS 24% longer while finding only about one positive every two days, a classic low-yield trade-off [25]. Our data show the same trap: a 120-minute COVID-19 test step made up almost half of the pre-intervention lead time. The takeaway is to manage testing with clear, adjustable rules, switch between targeted rapid tests and wider screening based on local rates and how patients are grouped. That switch is an operations tool as much as a clinical one. Recent studies support using these toggles on purpose, not by default [18].

Three design choices likely drove the size of the effect. First, we removed the biggest gate: a 120-minute wait. Many projects make small tweaks; we cut the main block. Studies on point-of-care testing show this kind of change lowers LOS right away [18]. Second, we moved bed assignment earlier and did pre-transfer tasks in parallel, simple Lean steps that favor flow over small local fixes. As in the ED Lean literature, we did not add many more “care minutes”; we cut waiting: value-added time dipped slightly (100 to 85 minutes), while non-value-added time dropped sharply (290 to 90 minutes) [26]. Third, we spread points of responsibility through nurses and daily debriefs. Even before a full EHR I-PASS, this “human glue” cut last-minute orders and made delays easier to see. That matches reports in the Joint Commission’s journal as clinicians wanted clear appointed responsibilities and standard steps for boarded patients [23].

Our results likely apply to hospitals with a similar Pareto pattern and basic IT visibility. In many settings, “Delay in receiving unit” and “Bed not ready” are the top issues; across systems, bed access and coordination have the biggest impact on both ED and inpatient LOS [4]. Some hospitals have different bottlenecks, such as lots of behavioral-health boarding or too few single rooms. In those cases, start with targeted testing and set aside inpatient beds for high-boarding services. Even without fancy dashboards, simple tracking boards and standard checklists can make a real difference when used consistently [27].

Implications for policy and practice

The implications of this study are best thought of as local, context-specific lessons from a local quality-improvement experience. In this context, earlier coordination of bed placement, attention to delays in receiving units and bed-readiness, and review of steps in transfer-gating were found to be associated with shorter admission-cycle times. A practical consequence is the need for structured, system-level admission-flow management, with well-defined testing criteria, earlier decisions on bed placement, a well-defined handoff from ED to ward, and clear monitoring of transfer times. These strategies are consistent with evidence that boarding needs to be coordinated across the hospital and not just in the ED [20]. Although these results can be used to inform other hospitals in considering other potential targets for local pathway review, they should not be seen as universal prescriptions or as proof that any one element of the intervention package is effective in isolation.

Limitations in light of the literature

There are some limitations to take into account. First, as this was a pre-post quality-improvement study at a single center, causal attribution cannot be made. The effect may be due to temporal confounding, and secular trends, staffing changes, seasonal changes, changes in COVID-19 policies, or other changes in processes at the same time may have played a role. Second, as the redesign was implemented as a package intervention, it is not possible to determine the relative contribution of each component. Third, the testing loop as a baseline bottleneck is dominant in the current day; it may be less relevant in other contexts or in a post-pandemic world. Fourth, Pareto categories were determined from operational tallies, and there may have been misclassification between the delay sources: receiving-unit delay, bed-readiness delay, and last-minute orders. Fifth, pathway medians derived from the map and EHR-derived admission cycle times are complementary but not identical analytic perspectives, and should not be interpreted as directly interchangeable. Lastly, there was a lack of systematic collection of balancing measures such as ward workload, the utilization of porters, and overtime for environmental services, as well as patient-centered clinical outcomes.

Future directions grounded in evidence

The evidence suggests a clear way to keep and grow these gains: use rapid, targeted tests, and adjust testing rules based on local prevalence. This protects flow without hurting safety, backed by ED studies of point-of-care polymerase chain reaction and cautionary reports on universal testing [18]. A live dashboard showing admission order time, consult arrival, environmental services start/finish, bed-ready, and inpatient bed arrival would turn our map into real-time tracking. Studies show these displays improve timeliness when rules and ownership are clear [3,4]. Standardizing the boarding bundle, with an EHR-embedded I-PASS handoff and scheduled reassessments, targets the quality problems repeatedly documented in qualitative studies and organizational guidance [23]. Further, because harm concentrates in the tail, track both averages and the longest waits, with special focus on older adults. Overnight ED stays are linked to higher mortality, and delirium risk rises with each extra hour of boarding [17].

## Conclusions

This single-center, pre-post, quality-improvement study found that a bundled Lean redesign of the ED-to-inpatient admission pathway was associated with a significant decrease in the time to admission from the ED (390 to 175 minutes) and an increase in process efficiency (26% to 49%). The observed gains seemed to be largely due to reduced non-value-added delay, such as the removal or streamlining of a well-known COVID-19 testing transfer gate and better coordination downstream. The COVID-19-related bottleneck was a particular operational situation of the pandemic, and the size of the benefit may not be directly transferable to other situations without similar transfer needs. Because of the study design, it is not possible to draw firm conclusions about cause and effect, or to attribute the effect to any one component of the intervention. Additional multi-center and time-series evaluations are required to determine the durability, generalizability, and the relative impact of the various components of the intervention package.
